# Early- to Mid-Term Review of a Prospective, Multi-Center, International, Outcomes Study of an Anatomically Designed Implant with Posterior-Stabilized Bearing in Total Knee Arthroplasty

**DOI:** 10.3390/medicina59122105

**Published:** 2023-11-30

**Authors:** Sung Eun Kim, Du Hyun Ro, Myung Chul Lee, Jason M. Cholewa

**Affiliations:** 1 Department of Orthopedic Surgery, Seoul National University Hospital, 101 Daehak-Ro, Jon Gno-Gu, Seoul 03080, Republic of Korea; paxgospel@gmail.com (S.E.K.); leemc@snu.ac.kr (M.C.L.); 2 Zimmer Biomet, 1800 W Center St., Warsaw, IN 46581, USA; jason.cholewa@zimmerbiomet.com

**Keywords:** posterior stabilized, aseptic loosening, knee replacement, knee society score

## Abstract

*Background and Objectives:* National joint registries report higher total knee arthroplasty (TKA) revision rates in posterior-stabilized (PS) systems compared to non-posterior-stabilized designs. The purpose of this study was to investigate the implant survivorship and clinical outcomes of an anatomic implant with a PS bearing. *Materials and Methods:* An early- to mid-term follow-up of a prospective, multi-center, non-controlled outcomes study of patients who received primary TKA between November 2014 and June 2017 was performed. A total of 800 cases using PS bearings that were implanted in 664 patients were monitored post-operatively for their implant survivorship and adverse events for up to five years. The Knee Society Knee and Function scores, patient satisfaction, the five-dimensional European Quality of Life questionnaire, and range of motion (ROM) were evaluated pre-operatively and post-operatively at six weeks, six months, one year, two years, three years, and five years. *Results:* The mean follow-up period was 3.7 ± 1.3 years, and the three-year implant survival rate was 99.3% (95% CI: 98.4%, 99.7%) with five revisions during the five-year follow-up. Patient satisfaction was 96.1% at six weeks and increased to 99.3% at one year. All patient-reported outcome measures significantly (*p* < 0.0001) increased up to the one-year follow-up and then remained stable up to the five-year follow-up. *Conclusions:* This study supports the excellent survivorship and patient-reported outcomes of the Persona^®^ Knee system using cemented, fixed bearing, posterior-stabilized components with minimal complications at early- to mid-term follow-up in an international Asian population. Ongoing observations are being performed to investigate the mid- to long-term survivorship and clinical outcomes associated with this knee system.

## 1. Introduction

Total knee arthroplasty (TKA) is a common and highly effective musculoskeletal procedure for treating end-stage osteoarthritis (OA) [1]. With its success, the rate of primary TKA has increased by over 400% in the first decade of the 21st century in Korea [2], and the demand for TKA is expected to increase by up to 500% by 2050 in the United States [3]. While the incidence of TKA in patients older than 65 continues to grow and comprises the majority of TKAs [4], TKAs in adults under 65 have more than tripled in the first decade of the 21st century [5] and suggest a greater inclusivity of the TKA indication. 

Despite advancements in orthopedic devices and surgical techniques, patient dissatisfaction following TKA has been reported to be as high as 10–20% [6,7,8], and functional performance deficits (i.e., those associated with walking and stair-climbing speed and knee extensor and flexor strength) persist in patients for at least one year following TKA [9,10]. Additionally, the TKA revision rate is not expected to decline by 2030 [11], and aseptic loosening remains one of the main causes of revision, especially in periods greater than two years post-surgery [12,13]. An analysis of the American Joint Replacement Registry from 2012 to 2019 reported revisions due to aseptic loosening to be higher in posterior-stabilized (PS) bearing designs (1.49%) compared to non-posterior-stabilized designs (1.18%) [14], and the United Kingdom National Joint Registry (UK NJR) reported similar findings, with five-year all-cause revision rates for PS and unconstrained bearings of 2.42% and 1.86%, respectively [15]. Although PS bearings are often prescribed for patients with greater pre-operative deformities, Abdel [16] reported a significantly lower 15-year survival in PS TKAs compared to cruciate retaining TKAs when adjusting for age, sex, and pre-operative deformity.

The shift in TKA inclusivity to include younger and more active adults [17], in addition to rising OA prevalence, active lifestyles, obesity, and expected lifespan has prompted greater expectations of orthopedic component longevity, durability, and functionality by surgeons, health care providers, and patients. Certain limitations in knee design systems still require surgeons to accept compromises (i.e., selecting maximal tibial bone coverage or ideal rotational alignment) that may result in compromised clinical outcomes [18,19]. Anatomic components feature a more contoured anatomic shape and physiological composition designed to recreate natural kinematics that may address these requirements and enhance patient outcomes [20,21]. 

While the five-year survivorship of the PS anatomic knee system is similar to that reported by the Australian Orthopaedic Association National Joint Registry (AOANJRR: 97.3%) [22] or better (99.1%) [23] than other PS knee system survival rates (98.1%) [24], two recent studies have reported slightly higher revision rates due to the aseptic loosening associated with the shorter keels, especially in obese patients [25,26], and the UK NJR reports the five-year survival to be lower than average, at 96.7%. As a result of these mixed reports, a large cohort, multi-surgeon and multi-country study is necessary to further determine the revision etiology and survival rate of this PS anatomic implant. The purpose of this study was to analyze early- to mid-term implant survivorship and clinical outcomes following TKA with anatomic PS fixed bearing implants. Specifically, we sought to evaluate the three-year survivorship of the PS system studied in a primarily Asian population.

## 2. Materials and Methods

**Figure 1:**
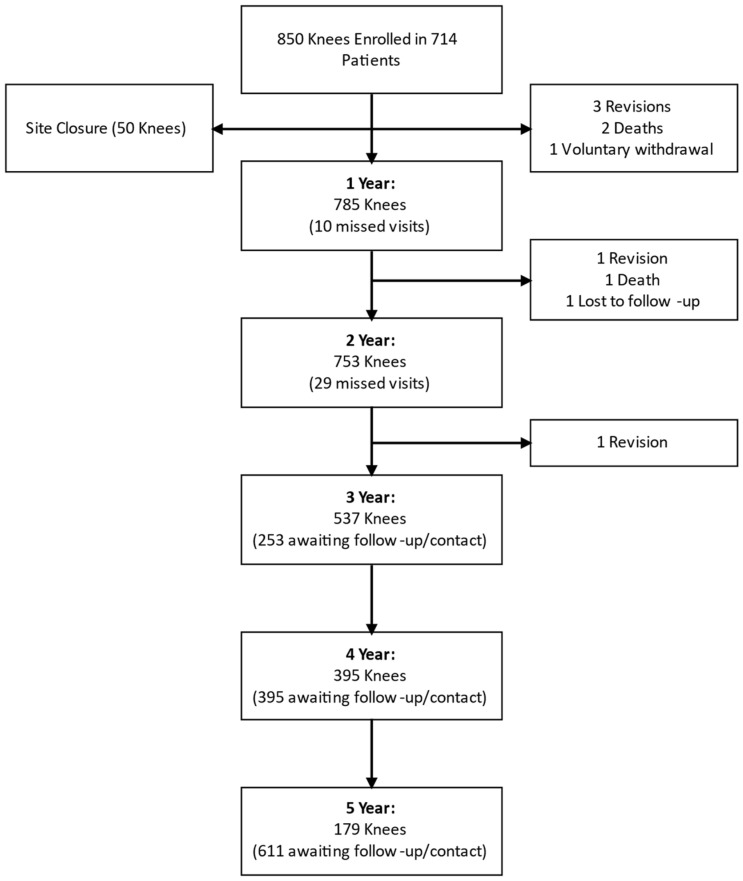
Strobe flowchart.

This was a prospective, multi-center, international, non-controlled outcomes study of patients who received primary TKA between November 2014 and June 2017 at 16 surgical centers in India (30.3%), Japan (12.2%), Korea (55.6%), New Zealand (0.3%), and Singapore (1.8%). To avoid a potential selection bias, participation was offered consecutively to eligible patients by each investigator. Patients who qualified for a primary TKA based upon the diagnosis of arthritis (rheumatoid, OA, traumatic, and polyarthritis), the avascular necrosis or collagen disorders of the femoral condyle, the post-traumatic loss of joint configuration, moderate deformity (varus, valgus, flexion), and/or the salvage of previously failed surgical attempts that did not include partial or full arthroplasty of the ipsilateral knee were eligible for participation. Patients with a history of infection in the affected joint or diseases that could influence bone metabolism were excluded from the study. Additional exclusion criteria included the following: participation in any other surgical intervention studies, an insufficient bone stock of the femoral or tibial surfaces, skeletal immaturity, the loss of musculature or neurovascular disease that compromises the affected limb, severe instability secondary to the absence of collateral ligament integrity, rheumatoid arthritis accompanied by an ulcer of the skin or a history of recurrent breakdown of the skin, and known or suspected sensitivity or allergy to one or more of the implant materials. One site closed in 2018 due to the departure of the primary investigator, and the 50 knees implanted at that site were excluded from the analysis, resulting in 800 cases available for review (Figure 1). Ethics approval for all sites was obtained (ClinicalTrials.gov Identifier: NCT04461626), and all subjects provided written informed consent prior to enrollment. 

All patients underwent TKA using fully cemented, fixed bearing, non-stemmed, PS total knee implants (Persona Knee System, Zimmer Biomet, Warsaw, IN, USA). Standard surgical procedure was followed under general or spinal anesthesia, according to the surgeon’s preference. Patellar resurfacing was indicated for patients presenting with an International Cartilage Repair Society Grade (ICRS) exceeding grade 2. Specifically, patellae with a minimum thickness of 12 mm at the thickest point were resurfaced, matching the thickness of the patellar component to mitigate the risk of post-operative patellar fracture. In cases where the patella was not resurfaced, any prominent osteophytes were removed. Surgical data collected included the type of anesthesia, ASA classification, surgical approach, intraoperative complications, and polyethylene component used. 

The primary outcome of this study, implant survival, was assessed by identifying any adverse events that required a revision of any component for any cause. We also reviewed any adverse events associated with the surgical limb. Secondary outcomes included the Knee Society Knee Score (KSS) and Function Score (KSFS), patient satisfaction (five-point Likert scale: very dissatisfied, dissatisfied, neutral, satisfied, very satisfied), the five-dimensional European Quality of Life (EQ5D-5L) questionnaire (index and visual analog scale [VAS]), and range of motion (ROM). The 2011 revised KSS [27] was administered to patients at sites in Japan (*n* = 98) and New Zealand (*n* = 2); however, due to language translation limitations, the original KSS [28] had to be administered to all other patients (*n* = 700). All follow-up measures were assessed pre-operatively and post-operatively at six weeks, six months, one year, two years, three years, and five years. 

All data are presented as means ± standard deviations, unless mentioned otherwise. Continuous data were evaluated with paired samples *t*-tests. Kaplan–Meier survivorship analyses were performed with any adverse events that required a revision of any component for any cause. Based on the results from the 2013 Australian Orthopaedic Association Joint Replacement Registry report referenced at the time of study initiation, the cumulative revision rate of primary TKA in osteoarthritis at five years is 3.8% or the survival rate is 96.2% [29]. The point estimate of this survival rate and its 95% two-sided confidence interval was produced. Based on an expected survival rate of 96.2% at five years, when the sample size is 328, a two-sided 95% confidence interval for the implant survival rate using the exact binomial confidence interval extended from 93.3% to 97.9%. Statistical significance was set to an α of *p* < 0.05 a priori, and all analyses were performed with SAS v9.4 (SAS Institute, Inc., Cary, NC, USA).

## 3. Results

A total of 800 primary TKAs were performed with a midvastus (*n* = 6), subvastus (*n* = 61), or medial parapatellar (*n* = 733) approach. The mean age of the population was 67.2 ± 7.5 years, the main indication for surgery was OA (97.1%) followed by rheumatoid arthritis (2.0%) and osteonecrosis (0.8%), and the majority of the population was female (Table 1). Femoral cement was used in 785 (98.1%) cases, and uncemented porous coated components were used in the remaining 15 cases. Vitamin E highly crosslinked polyethylene tibial inserts were used in 96 (12%) cases, and the patella was resurfaced in 120 (15%) cases. The mean operating room (OR) time was 90.6 ± 36.9 min for all patients, 94.7 ± 30.3 min for unilateral patients (*n* = 528), and 86.2 ± 46.2 min for bilateral cases (*n* = 136). 

**Figure 2:**
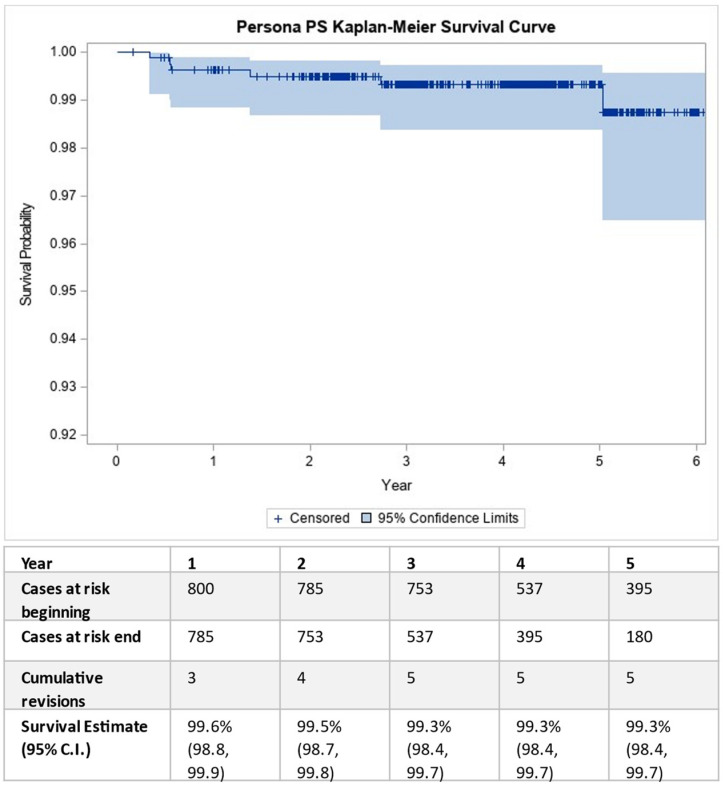
Kaplan–Meier survival curve.

The mean follow-up period was 3.7 ± 1.3 years. The implant survivorship at the three-year follow-up was 99.3% (95% CI: 98.3, 99.7%, Figure 2). There were five failures (0.63%) that led to revision surgery (Table 2) in the five-year follow-up period. There was an additional tibial implant loosening that required revision in the sixth year assessed to be related to the device by the investigator. There were five knee-related post-operative complications that did not result in revision surgery: one superficial joint infection resolved with wound revision, two patellar periprosthetic fractures, one case of patellar clunk syndrome that was treated with arthroscopic synovectomy, and one case of subsequent trauma that resulted in an MCL avulsion that was treated conservatively. 

The KSS and KSFS were significantly greater (*p* < 0.0001) at six weeks compared to those measured pre-operatively and increased through the first year before becoming stable (Table 3). The mean improvement in KSS and KSFS at six weeks were 35.3 (95% CI: 33.8, 36.8) and 6.3 (95% CI: 4.5, 8.2), respectively. At the one-year post-operative follow-up, the mean improvement in KSS and KSFS from the pre-operative measurement were 47.6 (95% CI: 46.2, 48.9) and 31.8 (95% CI: 30.3, 33.3), respectively. There were significant (*p* < 0.05) differences in KSS and KSFS, favoring un-resurfaced patellae through the first- and second-year post-operative periods, respectively. However, neither of the differences exceeded the MCID of nine points [30]. 

Significantly (*p* < 0.0001) more patients were satisfied with their operation than dissatisfied at the six-weeks post-operative follow-up, and this value remained high (98.3 to 99.3%) throughout the five-year post-operative period (Table 4). 

**Figure 3:**
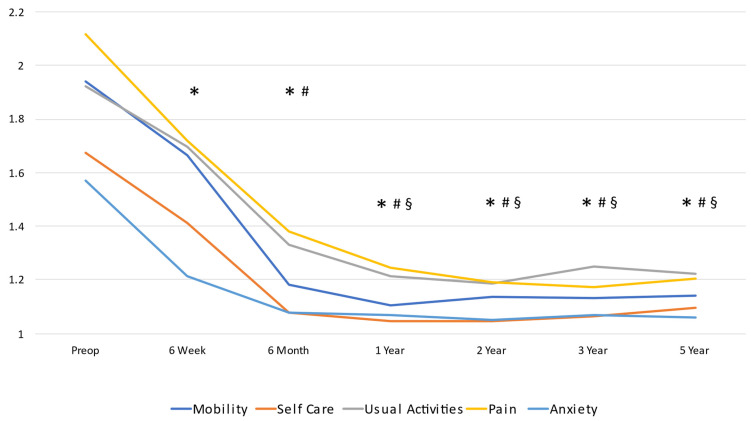
EQ5D-5L subscales. * significantly different from pre-operative assessment; # significantly different from six-week assessment; and § significantly different from six-month assessment.

Both EQ5D-5L index and EQ5D VAS significantly (*p* < 0.0001) improved at the six-week post-operative follow-up and continued to improve throughout the first year before becoming stable (Table 5 and Figure 3). The mean improvements in EQ5D-5L index and EQ5D VAS at six weeks were 0.196 (95% CI: 0.177, 0.215) and 13.3 (95% CI: 11.8, 14.8), respectively. At the one-year post-operative follow-up, the mean improvement in EQ5D-5L index and EQ5D VAS from the pre-operative measurement were 0.402 (95% CI: 0.384, 0.421) and 25.8 (95% CI: 24.4, 27.3), respectively.

**Figure 4:**
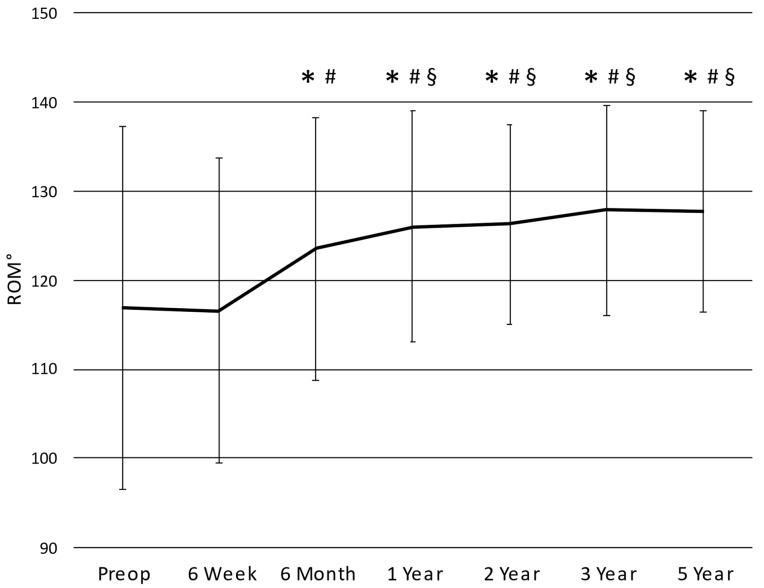
Arc of range of motion. * significantly different from pre-operative assessment; # significantly different from six-week assessment; and § significantly different from six-month assessment.

The extension ROM improved significantly (*p* < 0.0001) between the pre-operative period (5 ± 6.8°) and the six-week follow-up (1.9 ± 3.8°) and then stabilized after the first year (0.7 ± 2.3°). Pre-operatively, there were 202 cases (25.3%) of extension lag that showed a steady decrease in frequency through the follow-up period (Table 6). The flexion ROM was significantly lesser (*p* < 0.0001) at six weeks (118.5 ± 16.5°) compared to the pre-operative period (121.9 ± 17.5°) but recovered to levels greater than those during the pre-operative period at six months (124.5 ± 14.4°). The arc of ROM (Figure 4) showed a similar trend, increasing post-operative from six weeks to one year before stabilizing. 

## 4. Discussion

The most important findings of this study were that three- and five-year survivorship rates were 99.3% for this implant system, when failure was defined as a revision of any component for any reason. This is especially important as these data represent a large cohort of a primarily Asian population previously under reported in the TKA literature for this implant. 

The survivorship for this implant system found in the most recent Australian Orthopaedic Association National Joint Replacement Registry (AOANJRR) report [22], containing information on 4852 knees, was 97.8% at three years and 97.7% at five years. The UK National Joint Registry (NJR) [15], containing information on 2134 knees, reported a slightly lower five-year survival of 96.8% for the same system. Similarly, small cohort studies have reported three-year all-cause survivorship of 96.6% [23] and five-year survivorship from 97.8 to 98.7% [31,32] for the same device. The difference in our outcomes and those cited may be associated with the cohorts evaluated as well as the size of the cohorts. Regardless, our results are comparable, if not slightly greater, to other commercially available PS implants. Abdel et al. [24] reported a five-year all-cause revision rate of 96% for 5098 TKAs performed with PS components between 2000 and 2012. The AOANJRR reported three- and five-year survival rates for primary total knee arthroplasty due to osteoarthritis of 97.8% and 97.3%, respectively. The three-year survivorship of other fully cemented PS bearings in the AOANJRR 2022 annual report ranged from 97.2 to 96.3% [22]. Whereas the UK NJR reported an overall three-year survivorship for fully cemented PS, fixed, bearings of 98.4%, dependent upon age and sex at time of the index procedure. Looking at our study population as a whole, our data are consistent with the UK registry when compared to the age and sex categories as the mean age of our population was 67.2 ± 7.5 and we had a preponderance of female cases. For patients aged 65–74, in the UK NKR annual report, the survival rates at three years were reported to be 98.33% for males and 98.61% for females [15]. It is also worth noting that 15% of patellae were resurfaced in the present study. In general, un-resurfaced PS knees appeared to perform worse than those with initial patellar resurfacing as per national joint registries [15], but this largely un-resurfaced group in the present study exhibited excellent early survivorship and PROMs.

There were six revisions in the present study, only two of which were due to aseptic loosening. These results contradict those of Garceau et al. [25] who reported a higher rate of revision due to aseptic loosening with the same anatomic PS implants but without the addition of a short tibial stem. In their study, the five-year survival rate in the stemless group was 94.5% compared to 100% in the stemmed group. The authors conducted a sub-group analysis based on the presence of obesity (defined as BMI > 40 kg/m^2^) and found a significantly lower (71.4%) survival rate in the stemless group. While these results are concerning, they are based off a small sample size of a total of 74 patients in the stemless group with only eight patients in the stemless obese group and are not in agreement with the larger body of literature, including the larger cohort in the present study.

Patient-reported outcome measures (PROMs) significantly improved in this study. Compared to pre-operative values, the Knee Society Knee Score (KSS) improved by an average of 34.5 points and 47.6 points at the six-week and one-year follow-up, respectively. The six-week improvement in KSS exceeds the minimal important clinical difference (MCID) of 9 points, and the one-year improvement exceeds the substantial clinical benefit of 40 points [30]. The Knee Society Function Score (KSFS) also improved by 27.7 and 31.8 points in the first six months and one year, respectively, both exceeding the MCID of 10 points [30]. These results are comparable to other studies of the same knee system that showed scores, exceeding those of MCIDs, of 35.3 and 26.3 point changes for the KSKS and KSFS at two years, respectively [23], or substantial clinical benefits with a score of 49 for both the KSKS and KSFS at five years [31]. We also found significant improvements in quality of life through the first year that were maintained through the follow-up period. The EQ5D-5L index increased by 0.4 points, which exceeded the MCID, and satisfied the minimal important difference (SMID) change of 0.26 [33]. The improvement in EQ5D-5L of 80% found in this study is also comparable to the EQ-5D change reported in the UK NJR of 77.7% (0.35 points) [34]. 

This was an international study of Asian patients, and thus, the outcomes should be considered with this population in mind. There is a higher expectation to perform a greater flexion ROM in this population due to cultural and religious necessity to perform squatting, kneeling, and cross-legged sitting [35]. As such, a design that allows for up to 155° of knee flexion is ideal for this population [36]. The flexion ROM significantly improved by 9.1° up to the first-year follow-up in this study and then stabilized. The mean flexion ROM at one-year follow-up was 124.5°, which is in the 75th percentile based upon a recent clinical reference chart following primary TKA [37] and comparable to other studies investigating this same knee system [31,32]. Our ROM results are also comparable to other PS knee systems in Asian cohorts that have reported the mean one- to three-year flexion ROM between 110° and 135° and mean improvements in ROM between 1° and 24° [38,39,40,41,42,43,44,45]. However, caution should be taken when interpreting these results in the context of other studies. Comparing the ROM between cohorts is problematic given that pre-operative ROM is the strongest predictor of post-operative ROM [46], and patients with higher pre-operative ROMs experience lesser improvements in the ROM post-operatively compared to more restricted patients [47]. 

### Limitations

First, as not all patients have completed the full five-year follow-up, the five-year survival and PROMs data must be considered as preliminary and interpreted with caution. Regardless, 395 patients were at risk at the beginning of the 5th year, which was greater than the calculated necessary sample size of 328. Second, this study was limited to the evaluation of a fully cemented, fixed bearing, posterior-stabilized bearing in an anatomic knee system and did not contain a comparator group of another modern implant. Consequently, it is difficult to compare our results with the long-term outcomes of other implants. Third, these results may not be generalizable to the western population. Compared to western TKA patients, Asian patients are reported to have a lower body weight and present a higher proportion of varus constitutions (35% vs. 24.5%) [35]. Finally, the proportion of females (85.5%) to males (14.5%) is similar to the demographics reported for Korean TKA patients (1:11.6) (Kim 2015) but greater than the frequency reported by the Osteoarthritis Institute for TKA in western patients (59% female) [48] and may not be generalizable to populations with different gender ratios.

## 5. Conclusions

This study supports the excellent survivorship and PROMs of an anatomic system using fixed bearing, posterior-stabilized components and demonstrates the low incidences of aseptic loosening (0.3%) and complications at early- to mid-term follow-up in an international Asian population. These findings are of particular importance as this population is underrepresented in the TKA research and has greater ROM demands. Ongoing observations are being performed to investigate the mid- to long-term survivorship and clinical outcomes associated with this knee system.

## Figures and Tables

**Table 1:** Demographics.

	Mean ± SD	Range
Age	67.2 ± 7.5	20–80
Body Mass Index (kg/m^2^)	27.9 ± 4.5	19–47
Height (cm)	154.7 ± 7.4	139–179
Weight (kg)	66.7 ± 12	41–115
Gender, *n* = male (% male) *n* = female (% female)	116 (14.5%) 683 (85.5%)	
Follow-up (y)	3.7 ± 1.3	0.2–6.8

**Table 2:** Etiology of revision surgeries.

Complication	Cases (% Total Cases)	Revision Year (Range)
Tibial Implant Loosening	2 (0.1%)	4.5 (3.6)
Instability	1 (0.1%)	1
Deep Periprosthetic Joint Infection	3 (0.4%)	1.3 (1–2)

**Table 3:** Knee Society Scores.

	Original Knee Society Score		Revised Knee Society Score	
Visit	KSS ^Ŧ^	KSFS ^Ŧ^	KSS ^Ŧ^	KSFS ^Ŧ^
Pre-Operative	38.6 (16.8)	51.3 (17.2)	42.7 (18.4)	43.5 (17.4)
6 weeks	74.0 (14.7) *	57.7 (20.7) *	85.8 (10.8) *	56.2 (18.5) *
6 months	83.4 (11.9) *	78.6 (16.0) *	90.7 (10.0) *	66.4 (20.7) *
1 year	86.1 (11.1) *	82.9 (15.3) *	91.2 (10.9) *	70.5 (18.9) *
2 years	84.9 (10.9) *	84.0 (14.2) *	91.8 (12.7) *	73.8 (17.6) *
3 years	86.2 (9.4) *	81.9 (14.6) *	95.8 (5.9) *	76.9 (15.7) *
5 years	86.5 (10.1) *	84.7 (14.2) *	95.8 (7.7) *	73.1 (22.1) *

Values represent mean (SD); * significantly different from pre-operative assessment; ^Ŧ^ KSS: Knee Society Score; KSFS: Knee Society Function Score.

**Table 4:** Patient satisfaction associated with surgery results.

Visit	Satisfied	Dissatisfied
6 weeks	743 (96.1%)	30 (3.9%)
6 months	714 (99.3%)	5 (0.7%)
1 year	692 (99.3%)	5 (0.7%)
2 years	551 (98.9%)	6 (1.1%)
3 years	327 (98.8%)	4 (1.2%)
5 years	177 (98.3%)	3 (1.7%)

Values represent frequencies (%).

**Table 5:** EuroQol 5 Dimension 5 Level scores.

Visit	EQ5D-5L	EQ5D VAS ^Ŧ^ Health State
Pre-operative	0.5 (0.2)	56.5 (17)
6 weeks	0.7 (0.2) *	69.6 (14.4) *
6 months	0.9 (0.1) *	78.1 (12.6) *
1 year	0.9 (0.1) *	82.1 (12.2) *
2 years	0.9 (0.1) *	84.4 (11.7) *
3 years	0.9 (0.1) *	82.3 (15.1) *
5 years	0.9 (0.2) *	74.8 (24.5) *

Values represent mean (SD); * significantly different from pre-operative assessment; ^Ŧ^ VAS: visual analog scale.

**Table 6:** Extension lag.

Visit	Frequency (%)	Mean (SD)
Pre-operative	202 (25.3%)	8.1 (4.8)
six weeks	105 (13.2)	7.3 (3.6)
six months	49 (6.7%)	6.9 (3.5)
one year	32 (4.6%)	7.1 (4.2)
two years	23 (4.1%)	6.6 (3.4)
three years	10 (3.0%)	5.4 (2.7)
five years	1 (0.6%)	10 (NA)

## Data Availability

The data are unavailable due to privacy reasons.

## References

[1] Steinhaus M.E., Christ A.B., Cross M.B. (2017). Total Knee Arthroplasty for Knee Osteoarthritis: Support for a Foregone Conclusion?. HSS J..

[2] Koh I.J., Kim T.K., Chang C.B., Cho H.J., In Y. (2013). Trends in use of total knee arthroplasty in Korea from 2001 to 2010. Clin. Orthop. Relat. Res..

[3] Inacio MC S., Paxton E.W., Graves S.E., Namba R.S., Nemes S. (2017). Projected increase in total knee arthroplasty in the United States—An alternative projection model. Osteoarthr. Cartil..

[4] Bernstein J., Derman P. (2014). Dramatic increase in total knee replacement utilization rates cannot be fully explained by a disproportionate increase among younger patients. Orthopedics.

[5] Losina E., Katz J.N. (2012). Total knee arthroplasty on the rise in younger patients: Are we sure that past performance will guarantee future success?. Arthritis Rheum..

[6] Choi Y.J., Ra H.J. (2016). Patient Satisfaction after Total Knee Arthroplasty. Knee Surg. Relat. Res..

[7] Gunaratne R., Pratt D.N., Banda J., Fick D.P., Khan R.J., Robertson B.W. (2017). Patient Dissatisfaction Following Total Knee Arthroplasty: A Systematic Review of the Literature. J. Arthroplast..

[8] Lin Y., Chen X., Li L., Li Z., Zhang Y., Fan P. (2020). Comparison of Patient Satisfaction Between Medial Pivot Prostheses and Posterior-Stabilized Prostheses in Total Knee Arthroplasty. Orthop. Surg..

[9] Naili J.E., Iversen M.D., Esbjörnsson A.-C., Hedström M., Schwartz M.H., Häger C.K., Broström E.W. (2017). Deficits in functional performance and gait one year after total knee arthroplasty despite improved self-reported function. Knee Surg. Sports Traumatol. Arthrosc..

[10] Valtonen A., Pöyhönen T., Heinonen A., Sipilä S. (2009). Muscle deficits persist after unilateral knee replacement and have implications for rehabilitation. Phys. Ther..

[11] Schwartz A.M., Farley K.X., Guild G.N., Bradbury T.L. (2020). Projections and Epidemiology of Revision Hip and Knee Arthroplasty in the United States to 2030. J. Arthroplast..

[12] Arsoy D., Pagnano M.W., Lewallen D.G., Hanssen A.D., Sierra R.J. (2013). Aseptic tibial debonding as a cause of early failure in a modern total knee arthroplasty design. Clin. Orthop. Relat. Res..

[13] Koh I.J., Cho W.-S., Choi N.Y., Kim T.K., The Kleos Korea Research Group (2014). Causes, risk factors, and trends in failures after TKA in Korea over the past 5 years: A multicenter study. Clin. Orthop. Relat. Res..

[14] Kendall J., Pelt C.E., Imlay B., Yep P., Mullen K., Kagan R. (2022). Revision Risk for Total Knee Arthroplasty Polyethylene Designs in Patients 65 Years of Age or Older: An Analysis from the American Joint Replacement Registry. J. Bone Jt. Surg. Am..

[15] Ben-Shlomo Y., Emma A.B., Clark K., Deere J., Evans C., Gregson T., Jones A., Judge E., Lenguerrand E., Marques M. (2023). The National Joint Registry 20th Annual Report 2023. https://reports.njrcentre.org.uk/Portals/0/PDFdownloads/NJR%2020th%20Annual%20Report%202023.pdf.

[16] Abdel M.P., Morrey M.E., Jensen M.R., Morrey B.F. (2011). Increased long-term survival of posterior cruciate-retaining versus posterior cruciate-stabilizing total knee replacements. J. Bone Jt. Surg. Am..

[17] Maradit Kremers H., Larson D.R., Crowson C.S., Kremers W.K., Washington R.E., Steiner C.A., Jiranek W.A., Berry D.J. (2015). Prevalence of Total Hip and Knee Replacement in the United States. J. Bone Jt. Surg. Am..

[18] Dall’Oca C., Ricci M., Vecchini E., Giannini N., Lamberti D., Tromponi C., Magnan B. (2017). Evolution of TKA design. Acta Biomed..

[19] Lemaire P., Pioletti D.P., Meyer F.-M., Meuli R., Dörfl J., Leyvraz P.-F. (1997). Tibial component positioning in total knee arthroplasty: Bone coverage and extensor apparatus alignment. Knee Surg. Sports Traumatol. Arthrosc..

[20] Dai Y., Scuderi G.R., Bischoff J.E., Bertin K., Tarabichi S., Rajgopal A. (2014). Anatomic tibial component design can increase tibial coverage and rotational alignment accuracy: A comparison of six contemporary designs. Knee Surg. Sports Traumatol. Arthrosc..

[21] Galea V.P., Botros M.A., Madanat R., Nielsen C.S., Bragdon C. (2019). Promising early outcomes of a novel anatomic knee system. Knee Surg. Sports Traumatol. Arthrosc..

[22] Smith P.N., McAuliffe G.D., McDougall M.J., Stoney C., Vertullo J.D., Wall C.J., Corfield P.R., Cuthbert S., Du A.R., Harries P. (2023). Hip, Knee and Shoulder Arthroplasty: 2023 Annual Report. Australian Orthopaedic Association National Joint Replacement Registry.

[23] Yang J., Heckmann N.D., Nahhas C.R., Salzano M.B., Ruzich G.P., Jacobs J.J., Paprosky W.G., Rosenberg A.G., Nam D. (2021). Early outcomes of a modern cemented total knee arthroplasty: Is tibial loosening a concern?. Bone Jt. J..

[24] Abdel M.P., Ledford C.K., Kobic A., Taunton M.J., Hanssen A.D. (2017). Contemporary failure aetiologies of the primary, posterior-stabilised total knee arthroplasty. Bone Jt. J..

[25] Garceau S.P., Harris N.H., Felberbaum D.L., Teo G.M., Weinblatt A.I., Long W.J. (2020). Reduced Aseptic Loosening With Fully Cemented Short-Stemmed Tibial Components in Primary Cemented Total Knee Arthroplasty. J. Arthroplast..

[26] Garceau S.P., Pivec R., Teo G., Chisari E., Enns P.A., Weinblatt A.I., Aggarwal V.K., Austin M.S., Long W.J. (2022). Increased Rates of Tibial Aseptic Loosening in Primary Cemented Total Knee Arthroplasty With a Short Native Tibial Stem Design. J. Am. Acad. Orthop. Surg..

[27] Noble P.C., Scuderi G.R., Brekke A.C., Sikorskii A., Benjamin J.B., Lonner J.H., Chadha P., Daylamani D.A., Scott N.W., Bourne R.B. (2012). Development of a new Knee Society scoring system. Clin. Orthop. Relat. Res..

[28] Insall J.N., Dorr L.D., Scott R.D., Scott W.N. (1989). Rationale of the Knee Society clinical rating system. Clin. Orthop. Relat. Res..

[29] Australian Orthopaedic Association National Joint Replacement Registry (AOANJRR) (2022). Hip, Knee & Shoulder Arthroplasty: 2022 Annual Report. https://aoanjrr.sahmri.com/documents/10180/732916/AOA+2022+AR+Digital/f63ed890-36d0-c4b3-2e0b-7b63e2071b16.

[30] Lizaur-Utrilla A., Gonzalez-Parreño S., Martinez-Mendez D., Miralles-Muñoz F.A., Lopez-Prats F.A. (2020). Minimal clinically important differences and substantial clinical benefits for Knee Society Scores. Knee Surg. Sports Traumatol. Arthrosc..

[31] De Villeneuve F.B., Jacquet C., Puech S., Parratte S., Ollivier M., Argenson J.N. (2021). Minimum Five Years Follow-Up of Total Knee Arthroplasty Using Morphometric Implants in Patients With Osteoarthritis. J. Arthroplast..

[32] Gallego C.D., Fenoll I.B.M., Contreras J.L.P., Coronas F.J.M., Cal M.d.C.T.d.l., Martín J.M. (2022). Midterm results of a new personalized knee implant for total knee arthroplasty: Implant survivorship and patient-reported outcome after five years’ follow-up. Eur. J. Orthop. Surg. Traumatol..

[33] Conner-Spady B.L., Marshall D.A., Bohm E., Dunbar M.J., Noseworthy T.W. (2018). Comparing the validity and responsiveness of the EQ-5D-5L to the Oxford hip and knee scores and SF-12 in osteoarthritis patients 1 year following total joint replacement. Qual. Life Res..

[34] NJR-UK (2022). The National Joint Registry 19th Annual Report. https://reports.njrcentre.org.uk/Portals/0/PDFdownloads/NJR%2019th%20Annual%20Report%202022.pdf.

[35] Kim D.K., Seo M.C., Song S.J., Kim K.I. (2015). Are Korean Patients Different from other Ethnic Groups in Total Knee Arthroplasty?. Knee Surg. Relat. Res..

[36] Zimmer Biomet (2017). Persona: The Personalized Knee Story.

[37] Kittelson A.J., Elings J., Colborn K., Hoogeboom T.J., Christensen J.C., van Meeteren N.L.U., van Buuren S., Stevens-Lapsley J.E. (2020). Reference chart for knee flexion following total knee arthroplasty: A novel tool for monitoring postoperative recovery. BMC Musculoskelet. Disord..

[38] Cho S.-D., Youm Y.-S., Park K.-B. (2011). Three- to six-year follow-up results after high-flexion total knee arthroplasty: Can we allow passive deep knee bending?. Knee Surg. Sports Traumatol. Arthrosc..

[39] Han H.S., Kang S.B. (2013). Brief followup report: Does high-flexion total knee arthroplasty allow deep flexion safely in Asian patients?. Clin. Orthop. Relat. Res..

[40] Hosaka K., Saito S., Ishii T., Mori S., Sumino T., Tokuhashi Y. (2011). Asian-specific total knee system: 5–14 year follow-up study. BMC Musculoskelet. Disord..

[41] Kim Y.H., Park J.W., Kim J.S. (2012). High-flexion total knee arthroplasty: Survivorship and prevalence of osteolysis: Results after a minimum of ten years of follow-up. J. Bone Jt. Surg. Am..

[42] Malik A., Salas A., Ben Ari J., Ma Y., Della Valle A.G. (2010). Range of motion and function are similar in patients undergoing TKA with posterior stabilised and high-flexion inserts. Int. Orthop..

[43] Nakagawa Y., Koga H., Nakamura T., Horie M., Katagiri H., Ozeki N., Ohara T., Sekiya I., Muneta T., Watanabe T. (2022). Mid-term clinical outcomes of a posterior stabilized total knee prosthesis for Japanese patients: A minimum follow-up of 5 years. J. Orthop. Sci..

[44] Shi W., Jiang Y., Wang C., Zhang H., Wang Y., Li T. (2020). Comparative study on mid- and long-term clinical effects of medial pivot prosthesis and posterior-stabilized prosthesis after total knee arthroplasty. J. Orthop. Surg. Res..

[45] Suggs J.F., Hanson G.R., Park S.E., Moynihan A.L., Li G. (2008). Patient function after a posterior stabilizing total knee arthroplasty: Cam–post engagement and knee kinematics. Knee Surg. Sports Traumatol. Arthrosc..

[46] Sahu N.K., Patnaik S., Nanda S.N., Jain M. (2019). Variables Determining the Postoperative Knee Range of Motion Following Cruciate-substituting Total Knee Replacement: A Prospective Study. Cureus.

[47] Rowe P.J., Myles C.M., Nutton R. (2005). The effect of total knee arthroplasty on joint movement during functional activities and joint range of motion with particular regard to higher flexion users. J. Orthop. Surg..

[48] Collins J.E., Deshpande B.R., Katz J.N., Losina E. (2016). Race- and Sex-Specific Incidence Rates and Predictors of Total Knee Arthroplasty: Seven-Year Data From the Osteoarthritis Initiative. Arthritis Care Res..

